# Supplementary material for: Age-Dependent Dysregulation of APP in Neuronal and Skin Cells from Fragile X Individuals
Source: Cells. 2023 Feb 27;12(5):758. doi: 10.3390/cells12050758 (PMC10000963; doi:10.3390/cells12050758)
Supplement: Supplementary file 1 [file cells-12-00758-s001.zip › cells-2048328 Supplementary Materials.pdf]

Supplementary Materials

Age-Dependent Dysregulation of APP in Neuronal and Skin Cells from Fragile X Individuals

Giulia Cencelli [1,2], Laura Pacini [1,3], Anastasia De Luca [1,4], Ilenia Messia [1], Antonietta Gentile [1,5], Yunhee Kang [6], Veronica Nobile [7], Elisabetta Tabolacci [7], Peng Jin [6], Maria Giulia Farace [1] and Claudia Bagni [1,8,*]

Supplementary Figures

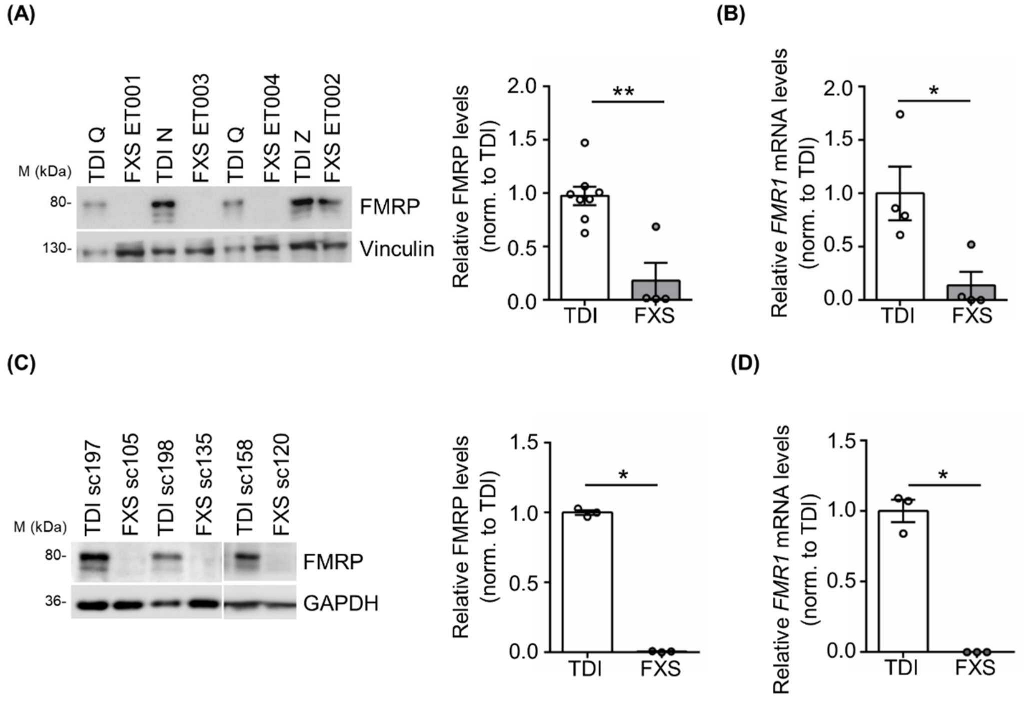

Figure S1. FMRP and *FMR1* mRNA levels in human fibroblasts and iPSCs. **(A)** Left, representative Western blot showing FMRP levels in TDI and FXS fibroblasts. Right, the bar plot shows FMRP protein quantification normalized to Vinculin (TDI $n = 8$; FXS $n = 4$). **(B)** The bar plot shows *FMR1* mRNA quantification in TDI and FXS fibroblast cell lines normalized to *HPRT1* and *GAPDH* mRNA levels (TDI $n = 4$; FXS $n = 4$). **(C)** Left, representative Western blot showing FMRP levels in TDI and FXS iPSCs normalized to GAPDH. Right, the bar plot shows FMRP protein quantification normalized to GAPDH (TDI $n = 3$; FXS $n = 3$). **(D)** The bar plot shows *FMR1* mRNA quantification in TDI and FXS iPSCs normalized to *HPRT1* and *GAPDH* mRNA levels (TDI $n = 3$; FXS $n = 3$). Error bars represent the SEM (* $p < 0.05$, ** $p < 0.01$ Mann-Whitney test).

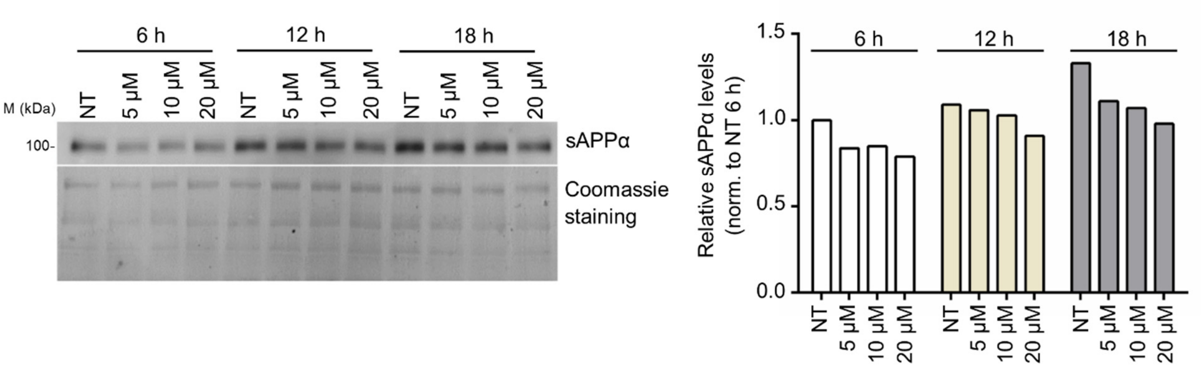

Figure S2. Time course of the TAT-Pro peptide treatment in FXS fibroblasts. Left, representative Western blot showing sAPPα levels in FXS fibroblasts treated with different peptide concentrations (5, 10 and 20 μM; NT, non-treated) for different time periods (6 h, 12 h and 18 h). Right, the bar plot shows the quantification of sAPPα normalized to Coomassie staining (average of technical duplicates).

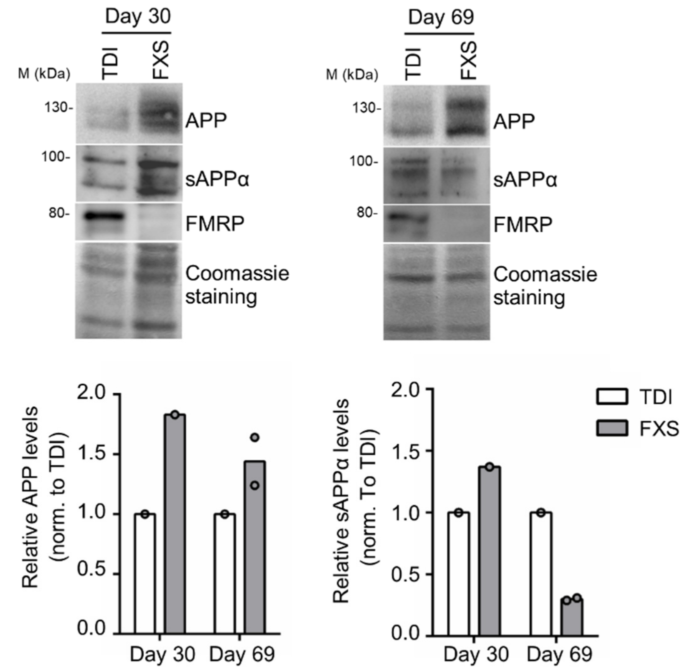

Figure S3. sAPPα levels in human forebrain organoids at two stages**.** Upper panels, representative Western blots showing the expression of APP, sAPPα and FMRP in human forebrain organoids at two different days in culture (day 30 and day 69) (TDI $n = 1$; FXS $n = 1$, technical duplicates). Lower panels, bar plots show the protein quantification normalized to Coomassie staining.

Supplementary Tables

Table S1: Fibroblast cell lines used in this study

Centers	ID	Gender	Age	*FMR1* mutation status	*FMR1* CGG repeat size	% Meth
Cohort in Switzerland (University Hospital CHUV, Lausanne)	NV00001*	M	38	Full mutation	340, 720, 1020	100%
	NV00002*	M	35	Mosaic	240, 300, 1150 (124)	> 95%
	NV00005*	M	38	Full mutation	310, 995	-
	NV00008*	M	23	Mosaic	260, 510, 770, 1030 (121)	> 95%
	NV00010*	M	37	Full mutation	345, 540, 700	100%
	NV00012*	M	17	Mosaic	240, 300 (185)	> 95%
	NV00013*	M	13	Full mutation	450, 570	-
	NV00014*	M	18	Full mutation	330, 575	-
	NV00017*	M	12	Mosaic	350, 740, 1020 (133)	> 95%
Cohort in USA (M.I.N.D. Institute, Sacramento)	1001-10-BZ*	M	13	Mosaic	390, 490, 950, 1140 (160-230)	94%
	1017-09-MR*	M	21	Mosaic	190, 220, 400, 500, 800 (65)	49%
	1022-10-ER*	M	22	Mosaic	235, 350, 650 (smear)	82%
	1043-10-SS*	M	23	Full mutation	290, 540, 790 light smear	100%
	1055-10-TG	M	12	Mosaic	255 (260-380)	12%
	TS114-12-KB*	M	15	Mosaic	230, 330 (30-200)	18%
	1020-09-SM*	M	24	Mosaic	180 (200-270)	16%
	1027-09-BZ*	M	32	Full mutation	510, 590, 670, 810	100%
	1027-11-ZD*	M	27	Full mutation	370	100%
	1032-10-SF	M	55	Mosaic	200 (250-760)	21%
	1035-09-LD	M	69	Mosaic	290 (200-250)	78%
	1060-10-GK*	M	29	Mosaic	425, 580, 680 (210-280)	79%
	TS106-13-JS	M	25	Full mutation	500, 820, 960	100%
Cohort in The Netherlands (Erasmus Medical Center, Rotterdam)	81E0152	M	8	Full mutation	490, 730	100%
	92E0198	M	6	Mosaic	200, (25)	> 95%
	84E0275	M	23	Full mutation	500	100%
	86E1377	M	32	Mosaic	410 (smear)	> 95%
	94E0363	M	19	Mosaic	> 200 (smear)	> 95%
	86E0681	M	22	Fragile X Syndrome[$]	> 200	-

Cohort in Italy (University Hospital A. Gemelli, Rome)	ET001	M	46	Full mutation	~630-790	100%
	ET002	M	31	Mosaic	95- >200	3%-100%
	ET003	M	42	Full mutation	~360-560	100%
	ET004	M	28	Full mutation	~360	100%
Control Cohort (Coriell Cell Repositories)	GM23973_H	M	19	-	-	-
	GM03440_F	M	20	-	-	-
	GM23964_R	M	21	-	-	-
	GM23976_O	M	22	-	-	-
	GM23815_K	M	22	-	-	-
	GM00500_J	M	10	-	-	-
	GM09503_C	M	10	-	-	-
	GM01864_G	M	11	-	-	-
	GM00316_P	M	12	-	-	-
	GM23971_D	M	33	-	-	-
	GM02673_E	M	33	-	-	-
	GM00409_B	M	7	-	-	-
	GM05381_M	M	5	-	-	-
	GM05400_N	M	6	-	-	-
	GM07492_Q	M	17	-	-	-
	GM08398_S	M	8	-	-	-
	GM13335_T	M	57	-	-	-
	GM23968_V	M	43	-	-	-
	GM23975_Z	M	25	-	-	-

$ Determined cytogenetically as Fragile X.
* FXS fibroblast cell lines used for the correlation analysis between the main domains of Vineland adaptive behavior scale and sAPPα levels.

Table S2: Human iPS cell lines used in this study from Children's Hospital of Orange County, USA

	ID	Gender	Age	*FMR1* mutation status	*FMR1* CGG repeat size
FXS	SC105	M	12	Mosaic	440, 710, 900, 1100 (260)
	SC120	M	21	Mosaic	475, 625, 810 (~250)
	SC135	M	20	Full mutation	450
TDI	SC158	M	29	-	-
	SC197	M	60	-	-
	SC198	M	20	-	-
